# Association between myopia progression and cylinder power progression in defocus incorporated multiple segments lens wearers

**DOI:** 10.1038/s41598-025-10099-7

**Published:** 2025-07-06

**Authors:** Ling Wang, Qing Zhang, Tingting Zhou, Wenli Yu, Jincheng Wang, Ran Li, Qin Chen, Qingbo Que, Xiaomin Gao

**Affiliations:** 1 Department of Optometry Engineering, Jinling Institute of Technology, Nanjing, 211169 China; 2 Ophthalmology Department, Nanjing Tongren Hospital, School of Medicine, Southeast University, Nanjing, 210000 China

**Keywords:** Cylinder power, Axial length, Astigmatism, Cornea astigmatism, Myopia, DIMS, Health care, Medical research

## Abstract

The aim of this study was to explore the correlation between the parameters associated with the prevention and management of myopia and cylinder power (CP) progression in individuals who wear defocus incorporated multiple segments (DIMS) lenses. In total, 179 children (6–12 years old) prescribed DIMS lenses for 1 year were enrolled in this retrospective cohort study. Cycloplegic refraction, spherical equivalent (SE), axial length (AL), CP, and corneal astigmatism (CA) were measured. The relationship between myopia and CP progression was assessed using binary logistic regression analyses, stratified analyses, and interaction tests. These 179 DIMS lens wearers were, on average, 9.51 ± 1.48 years old, and 51.4% were male. In total, 24.02% of these participants had CP progression ≥ 0.25 D within one year. A fully-adjusted binary logistic regression analysis revealed that ΔSE (odds ratio (OR) = 1.73, 95% CI (1.32, 2.27), *P* < 0.001) and CA at admission (OR = 1.17, 95% CI (1, 1.36), *P* = 0.049) were associated with CP progression following adjustment for potential confounding factors. Interaction analysis revealed no significant interactive relationships. Our results showed a significant positive association between ΔSE and CP progression in DIMS lens wearers. Furthermore, baseline CA was also associated with ΔCP in participants over the course of the study. This suggested that myopia progression and baseline CA levels contribute to variations in CP progression. This study helps optimize astigmatism control in preventing and controlling myopia in DIMS lens wearers.

## Introduction

Myopia is a global public health concern. Its prevalence has increased dramatically in recent decades, particularly in East Asian countries^1,2^. Astigmatism, resulting from the irregular refractive power of the eyeball in children and adults, is the most common refractive error, followed by hyperopia and myopia^3^. Astigmatism is primarily characterized by the degree of cylinder power (CP).

Although total astigmatism is the result of a combination of corneal astigmatism (CA) and ocular residual astigmatism^4^ optometrists and parents are more concerned with the change in total astigmatism, expressed as a change in CP when prescribing spectacles for myopia management, especially regarding defocus incorporated multiple segments (DIMS) lens wearers.

Studies have shown that myopia-controlled lenses are the least invasive and are safe for long-term use in many myopia control strategies^5^. As a myopia control spectacle, DIMS is a minimally invasive therapy to slow down myopia progression and has minimal negative consequences on the quality of life throughout the entire treatment, even at a young age. Many studies have confirmed the role of DIMS lenses in myopia prevention and control^6–11^. However, evidence that shows specific changes in astigmatism during the correction process using DIMS lenses is lacking. The identification of CP progression patterns associated with myopia control in children would facilitate a more comprehensive understanding of myopia control, thereby offering novel insights into the management of myopia.

The aim of this study was to explore the correlation between the parameters associated with the prevention and management of myopia and CP progression in individuals who wear DIMS lenses.The study analyzed the effect of wearing DIMS on changes in spherical equivalent refraction, CYL, and CA. This study divided the participants into two groups: CP unchanged or decrease group(ΔCP ≤ 0) and CP increased group(ΔCP ≥ 0.25D).

This study is the first to investigate the factors related to CP progression in myopia prevention and control in DIMS lens wearers.

## Methods

### Study design

This was a retrospective observational cohort study.

The study was conducted in accordance with the tenets of the Declaration of Helsinki, and approval was obtained from the Nanjing Tongren Hospital Affiliated to Southeast University School of Medicine (Ethics approval number: 2025-03-004-K001).

Electronic patient records were retrieved in the spring of 2025 for patients prescribed with DIMS and who consented to participate in the establishment of a refractive development profile.

Children and adolescents aged 6–12 years were enrolled and prescribed DIMS lenses (Hoya MiyoSmart, Tokyo, Japan) from July 2021 to August 2023. Cycloplegic refraction and axial length (AL) were assessed at baseline and 12 months as key outcome measures.

The Strengthening the Reporting of Observational Studies in Epidemiology guidelines were used for reporting.

### Inclusion and exclusion

Each patient consented to participate in establishing a refractive development profile, and follow-up examinations were completed at 6 and 12 months.

**Fig. 1:**
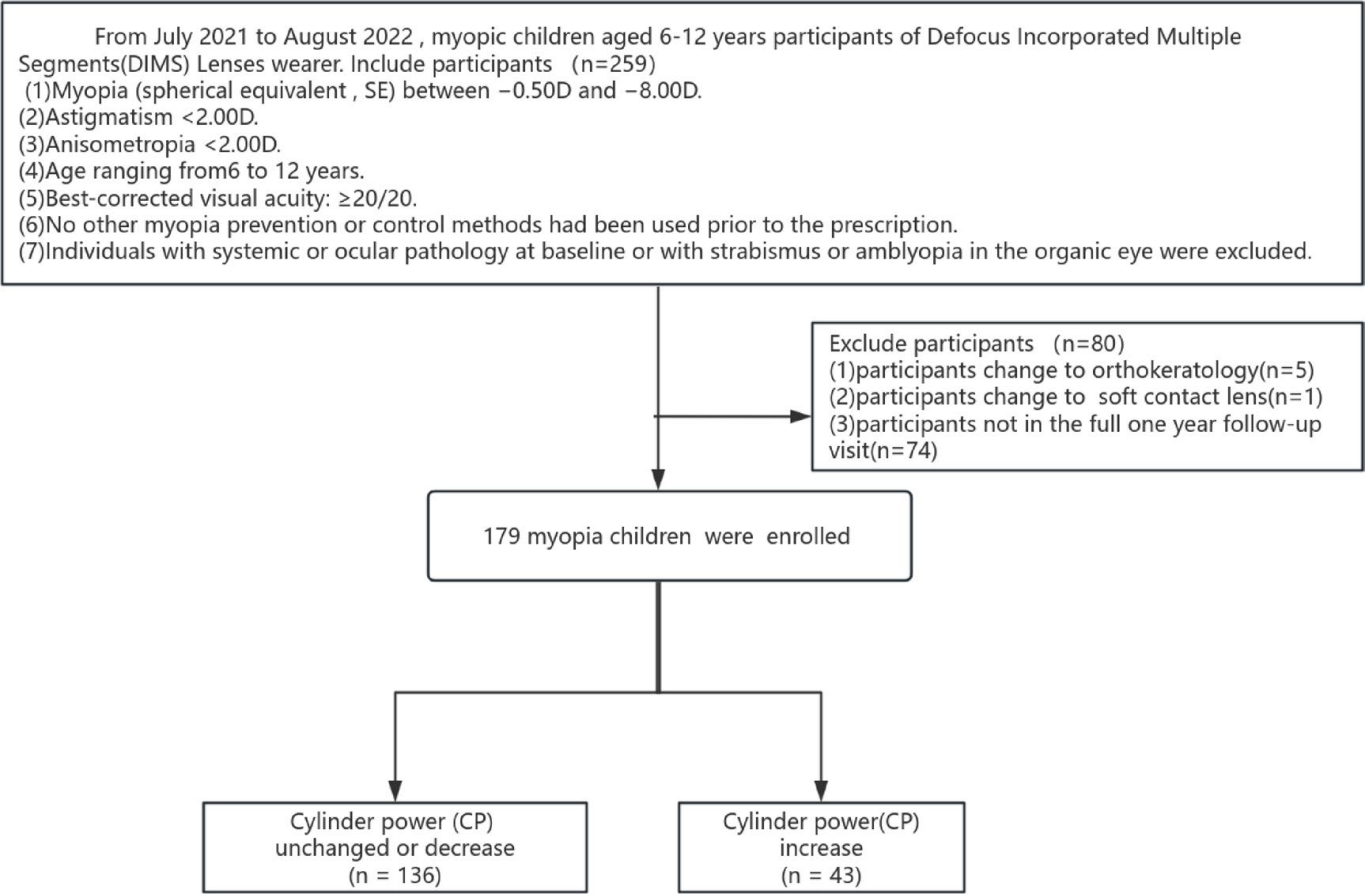
Flow diagram of inclusion and exclusion.

The flow diagram of inclusion and exclusion for participants is presented in Fig. 1.

The DIMS lens was replaced if there was a change of ≥ 0.50 D in the spherical equivalent (SE).

### Measurements

AL and refractive status (diopter of sphere (DS); diopter of cylinder (DC); CA, and AL/CR) were measured for all participants.

Refractive development profile were established for all patients. Each participant underwent a slit-lamp examination and intraocular pressure measurement to ensure the safety of cycloplegia.

AL and AL/CR were measured using an IOL Master 500 (Carl Zeiss, Germany) before DIMS lens wear and at each review. The measurements were repeated five times for each eye and averaged, and all AL measurements were performed before cycloplegia.

CA is defined as difference in refractive power between the two principal meridians of the cornea (i.e., the flattest keratometry value K1 and the steepest keratometry value K2) as measured by the IOL MASTER 500.after inducing cycloplegia. Three measurements for each eye were obtained, and the procedure was repeated if the difference between any two records of K1 or K2 was larger than 0.50 diopter (D).

An autorefractor (Topcon RM800, Japan) was used to perform objective refraction after inducing cycloplegia combined with subjective refraction to determine the prescription.

### Cycloplegic refraction and quality control

A compound tropicamide ophthalmic solution (0.5% tropicamide and 0.5% phenylephrine eye drops; zhuo bi an, xingqi, China)was administered to each eye, 1–2 drops per instillation, at 5-min intervals, for a total of three instillation. The pupillary light reflex and the diameter of the pupil were evaluated a minimum of 30 min after the final drop of the cycloplegic agent. The absence of light reflection and a pupil diameter greater than 6 mm were considered indicative of complete cycloplegia; otherwise, an additional drop of tropicamide was administered. It was imperative that the participants kept their eyes closed during the procedure to ensure completion. The longest time to complete refraction was 30–40 min after use.

An experienced optometrist conducted comprehensive optometry examination. The standard operating procedures of the refractive clinic have been implemented in our clinic at first visit. The clinician needs to perform retinoscopy, corneal topography, a slit-lamp examination and a retinal examination under small pupil.The treating ophthalmologists diagnosed keratoconus through the identification of typical clinical signs (scissors reflex, prominent corneal nerves, Vogt’s striae, corneal thinning or ectasia) and topographic findings.

All measurements were performed using the same equipment to minimize data discrepancies. A refractive development profile was established to identify and resolve problems related to myopia control. The database was reviewed by optometrists and the data management team to identify any missing or inaccurate information and to make the necessary corrections in a timely manner.

All instruments were calibrated before the examination.

### Definitions

#### Exposure variable

Referring to the ‘Expert Consensus on the Application of Spectacle Related to Myopia Prevention and Control in Myopia Management (2023)’^12^ a dual classification system was devised to categorize the definitions of myopia prevention and control effect.

The SE was calculated as DS + 0.5 × DC. In terms of ΔSE (SE control effect at 12 months). Group I was defined as those with a good response to myopia control after 12 months of lens wear and significant effect (ΔSE ≤ 0.25 D). Group II displayed an insignificant effect on myopia control (0.25 D < ΔSE < 0.75 D). Group III displayed a poor effect on myopia control (ΔSE ≥ 0.75 D).

In terms of ΔAL (AL control effect at 12 months), Group I displayed a good response to myopia prevention and control effect (ΔAL ≤ 0.20 mm).Group II displayed an effect of 0.20 mm < ΔAL ≤ 0.40 mm. Group III displayed a poor response in AL control (ΔAL > 0.40 mm).

#### Outcome variable

A change in DC over 1 year was defined as CP progression(ΔCP).children were classified by ΔCP.Clylinder power unchanged or decrease group(ΔCP ≤ 0)and Clylinder power increased group(ΔCP ≥ 0.25D).

#### Covariates

The children were divided into two groups according to their baseline age, i.e., participants aged 6–9 and 10–12 years.

The children was categorized into a low (− 3.00 D < SE ≤ − 0.50 D), moderate (− 6.00 D < SE ≤ − 3.00 D), or high (SE ≤ − 6.00 D) myopia group^13^.

### Statistical analysis

Owing to the high correlation between the right and left eyes, only data on the right eye were used for analysis in this study, and the Kolmogorov–Smirnov test was used to determine whether the variables were normally distributed. Normally distributed variables were presented as mean (standard deviation), while skewed variables were presented as median (interquartile range, 25–75%). Categorical variables were presented as proportions (%). Comparisons of continuous variables across groups were conducted using the independent samples Student’s *t*-test or Mann–Whitney U-test, contingent upon the normality of the distribution. Categorical data were compared using the χ2 test, as appropriate.

We used both univariate and multivariate binary logistic regression models to investigate the association between myopia and CP progression. Stratified analyses, interaction tests, and covariate screening were also performed. A multivariate single-imputation method for missing data based on an iterative imputer was implemented using R software (version 4.2.2; R Foundation for Statistical Computing; http://www. Rproject. org), and Free Statistics software (version 2.1; Beijing Free Clinical Medical Technology Co., Ltd.) was used for the analysis. Significance was set at a two-sided *P*-value < 0.05 for all analyses. Data were analyzed from March to April 2025.

## Results

### General characteristics

The demographics and characteristics of the wearers at the time of admission and at one year are summarized in Table 1. Of these patients, 87 were male and 92 were female, with a mean age of 9.51 ± 1.48 years. Furthermore, the CP of 24.02% of these participants exceeded 0.25 DC within 1 year.

**Table 1:** Baseline characteristics and one-Year changes in participants who wore DIMS lenses for one Year.

Variables	Overall	ΔCP		*P*-values
		CP unchanged or decrease (ΔCP ≤ 0) (*n* = 136)	CP increase (ΔCP ≥ 0.25D)(*n* = 43)	
Age, n (%)				0.639
6years	3 (1.68)	3 (2.21)	0 (0)	
7years	14 (7.82)	9 (6.62)	5 (11.63)	
8years	27 (15.08)	20 (14.71)	7 (16.28)	
9years	46 (25.70)	35 (25.74)	11 (25.58)	
10years	41 (22.91)	29 (21.32)	12 (27.91)	
11years	29 (16.20)	23 (16.91)	6 (13.95)	
12years	19 (10.61)	17 (12.5)	2 (4.65)	
Sex, n (%)				0.753
male	87 (48.60)	67 (49.26)	20 (46.51)	
female	92 (51.40)	69 (50.74)	23 (53.49)	
SE Baseline, Median (IQR), (D)	−2.50 (−3.50, −1.75)	−2.50 (−3.75, −1.75)	−2.62 (−3.25, −1.62)	0.847
Grade of SE Baseline, n (%)				0.937
Group I −3.00 D < SE≤−0.50 D(*n* = 105)	105 (58.66)	80 (58.82)	25 (58.14)	
Group II −6.00 D < SE≤−3.00 D(*n* = 66) And Group III SE≤−6.00 D(*n* = 8)	74 (41.34)	56 (41.18)	18 (41.86)	
AL Baseline, Mean ± SD, (mm)	24.70 ± 0.85	24.70 ± 0.86	24.72 ± 0.85	0.866
CP Baseline, Median (IQR), (DC)	−0.50 (−0.50, 0.00)	−0.50 (−0.50, 0.00)	−0.50 (−0.75, 0.00)	0.043
CA Baseline, Mean ± SD, (DC)	1.24 ± 0.63	1.19 ± 0.61	1.41 ± 0.66	0.053
AL/CR Baseline, Mean ± SD	3.13 ± 0.09	3.13 ± 0.09	3.12 ± 0.08	0.465
ΔSE(IQR), (D)	0.25 (0.00, 0.75)	0.25 (0.00, 0.50)	0.63 (0.25, 0.88)	< 0.001
Grade of ΔSE, n (%)				< 0.001
ΔSE ≤ 0.25 D	97 (54.19)	85 (62.5)	12 (27.91)	
0.25 < ΔSE < 0.75 D	34 (18.99)	24 (17.65)	10 (23.26)	
ΔSE ≥ 0.75 D	48 (26.82)	27 (19.85)	21 (48.84)	
ΔAL(IQR)	0.24 (0.11, 0.38)	0.24 (0.10, 0.34)	0.27 (0.12, 0.41)	0.336
Grade of Δ AL, n (%)				0.261
ΔAL ≤ 0.20 mm	74 (41.34)	57 (41.91)	17 (39.53)	
0.20 mm < ΔAL ≤ 0.40 mm	70 (39.11)	56 (41.18)	14 (32.56)	
ΔAL > 0.40 mm	35 (19.55)	23 (16.91)	12 (27.91)	
ΔCA, Median (IQR), (DC)	0.13 (0.00, 0.33)	0.11 (0.00, 0.33)	0.17 (−0.03, 0.35)	0.632
ΔCP, n (%)				< 0.001
−0.5DC	3 (1.68)	3 (2.21)	0 (0)	
−0.25DC	2 (1.12)	2 (1.47)	0 (0)	
0DC	131 (73.18)	131 (96.32)	0 (0)	
0.25DC	23 (12.85)	0 (0)	23 (53.49)	
0.5DC	19 (10.61)	0 (0)	19 (44.19)	
0.75DC	1 (0.56)	0 (0)	1 (2.33)	

DIMS: Defocus Incorporated Multiple Segments; AL/CR: Axial Length/Corneal Radius.

Plus-minus values are means ± SD. p values were calculated by comparing characteristics.

SE: spherical equivalent CA: Corneal astigmatism CP: Cylinder power D: diopter.

There were no significant differences in age, sex ratio, baseline SE, AL, or ΔAL between the groups with unchanged or decreased CP and those with increased CP (*P* > 0.05).

The SE level at admission was − 2.50 (− 3.50, − 1.75) D, and the ΔSE levels were significantly higher in wearers with increased CP than in those with unchanged or decreased CP (*P* < 0.001).

### Multivariate binary logistic regression models

ΔSE levels were predictive of ΔCP.

As shown in Table 2, increased SE levels were associated with an increased risk of CP. This analysis revealed that ΔSE was significantly associated with the incidence of ΔCP (odds ratio (OR) = 1.73,95% CI: 1.32–2.27). Every 0.25 D increase in ΔSE was associated with a 73% increase in the odds of ΔCP in DIMS lens wearers.

Table 2 shows a graded association between ΔSE and ΔCP risk (ODDS ratio for grade 3 ΔSE [ΔR-SE > 0.75 D] vs. grade 1 ΔSE [ΔR-SE ≤ 0.25 D] in the fully adjusted model, 6.32; (95% confidence interval [CI], 2.48 to 16.07).

We also found an association between CA baseline and ΔCP risk (1.17 (1.00–1.36). Every 0.25 D increase in CA baseline was associated with a 17% increase in the odds of ΔCP in DIMS lens wearers.

However, no statistically significant relationship was observed between ΔCP and ΔAL. The crude model showed an OR of 3.17 (95% CI: 0.47–21.13, *P* = 0.234), indicating no significant association between change in AL and myopia progression. This finding remained non-significant across all adjusted models (*P* > 0.05), with ORs ranging from 1.65 to 3.17.

### Stratified analysis

**Fig. 2:**
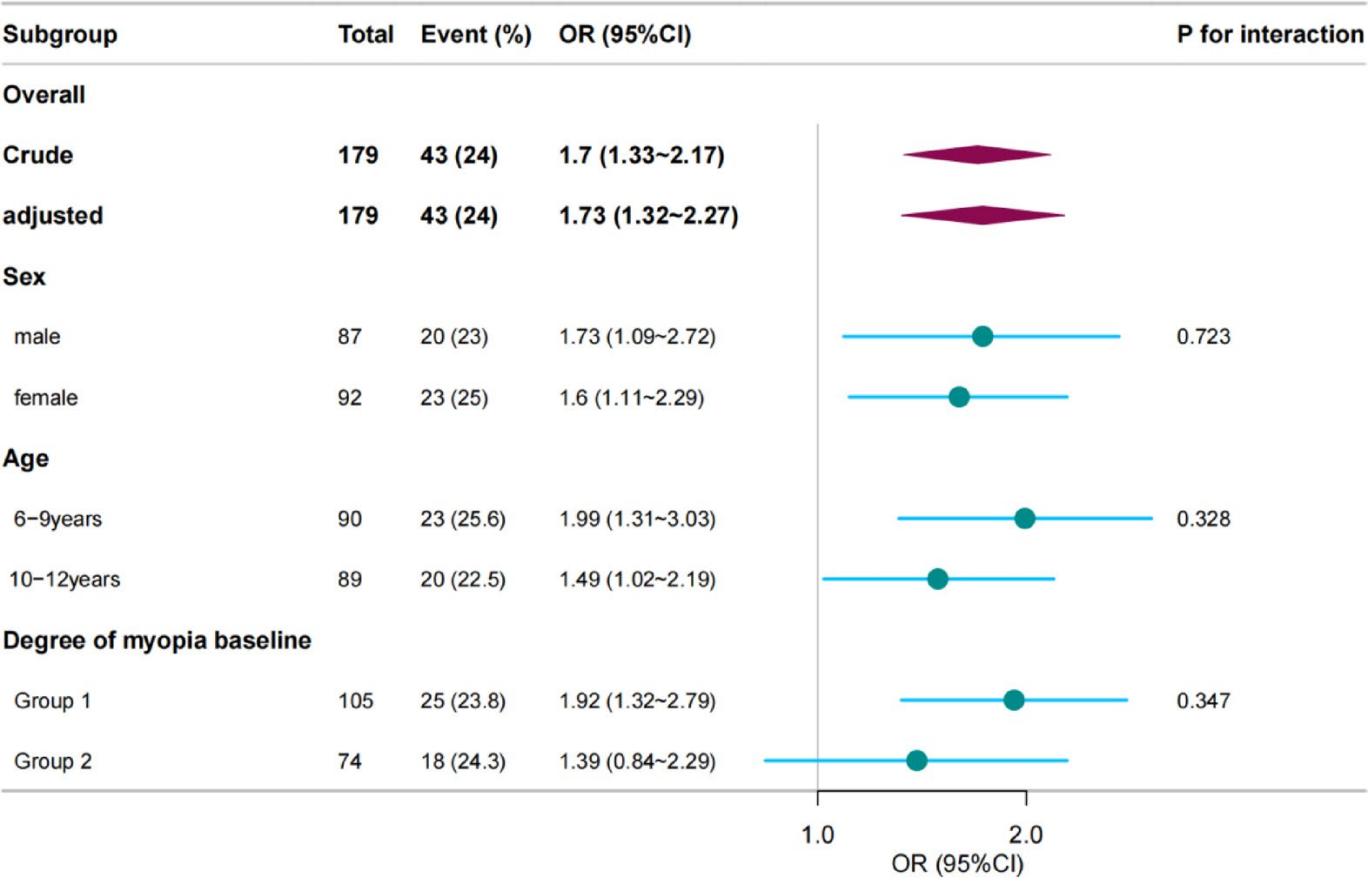
Stratified analysis of the association of ΔSE and CP progression in DIMS wearer. Group1: Group I −3.00 D < SE≤−0.50 D; Group2 : Group II −6.00 D < SE≤−3.00 D And Group III SE≤−6.00 D.

**Fig. 3:**
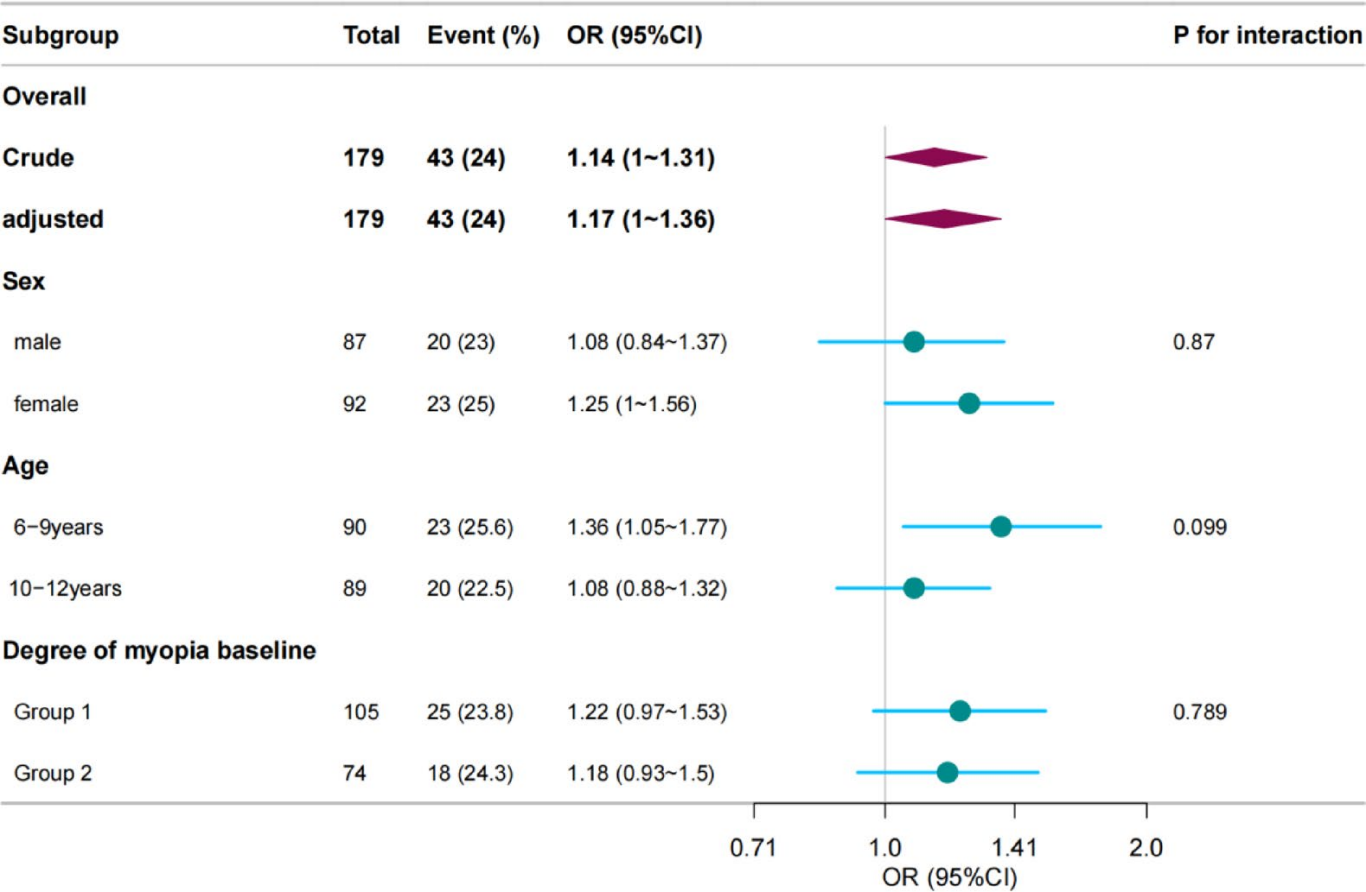
Stratified analysis of the association of baseline CA and CP progression in DIMS wearer. Group1: Group I−3.00 D < SE≤−0.50 D; Group2 : Group II −6.00 D < SE≤−3.00 D And Group III SE≤−6.00 D.

Forest plots show ORs in subgroup analyses. A stratified analysis yielded results consistent with those of our multivariate logistic regression analysis concerning the relationship between ΔSE and ΔCP incidence. In contrast, an interaction analysis detected no interactive association between ΔSE and ΔCP (Fig. 2). Also, no interactive association was detected between baseline CA and ΔCP (Fig. 3).

We conducted a subgroup analysis with baseline myopia to explore the influence of different baseline myopia groups on the association between ΔSE and ΔCP. The ORs in the two groups were similar, and no interaction was detected. In addition, the same subgroup findings were observed between baseline CA and ΔCP.

**Table 2:** Multivariable logistic regression analyses models evaluating the association between myopia progression and cylinder power progression.

Variable	N	Crude model		Adjusted model 1		Adjusted model 2		Adjusted model 3	
		OR (95%CI)	P -value	OR (95%CI)	P -value	OR (95%CI)	P -value	OR (95%CI)	P -value
ΔSE(0.25D)	179	1.7 (1.33~2.17)	<0.001	1.72 (1.32~2.25)	<0.001	1.72 (1.32~2.25)	<0.001	1.73 (1.32~2.27)	<0.001
Gade of ΔR-SE									
ΔSE≤0.25D	97	1(Ref)		1(Ref)		1(Ref)		1(Ref)	
0.25<ΔSE≤0.75D	34	2.95 (1.14~7.66)	0.026	2.82 (1.07~7.44)	0.036	2.84 (1.07~7.52)	0.035	2.82 (1.06~7.49)	0.038
ΔSE>0.75D	48	5.51 (2.4~12.65)	<0.001	6.15 (2.47~15.33)	<0.001	6.19 (2.48~15.49)	<0.001	6.32 (2.48~16.07)	<0.001
Trend.test		2.34 (1.55~3.53)	<0.001	2.48 (1.58~3.92)	<0.001	2.49 (1.58~3.93)	<0.001	2.52 (1.58~4.01)	<0.001
ΔAL(mm)	179	3.17 (0.47~21.13)	0.234	2.12 (0.26~17.54)	0.486	2.14 (0.25~18.1)	0.484	1.65 (0.18~14.91)	0.655
Grade of ΔAL									
ΔAL≤0.2mm	74	1(Ref)		1(Ref)		1(Ref)		1(Ref)	
0.20mm<ΔAL ≤ 0.40 mm	70	0.84 (0.38~1.86)	0.665	0.71 (0.31~1.65)	0.429	0.71 (0.31~1.65)	0.431	0.61 (0.25~1.47)	0.269
ΔAL>0.4mm	35	1.75 (0.72~4.23)	0.215	1.53 (0.57~4.12)	0.405	1.53 (0.56~4.15)	0.406	1.35 (0.48~3.85)	0.571
Trend.test		1.27 (0.8~1.99)	0.309	1.16 (0.7~1.91)	0.573	1.16 (0.7~1.92)	0.573	1.09 (0.64~1.85)	0.761
CA baseline(0.25DC)	179	1.14 (1~1.31)	0.056	1.16 (1~1.34)	0.047	1.16 (1~1.34)	0.047	1.17 (1~1.36)	0.049

The table presents the results of multivariate regression analyses examining the association between myopia progression and cylinder power progression.

Variables: CA baseline (D): Refers to the baseline cornea astigmatism in diopters. ΔSE (0.25D): Indicates the change in refractive spherical equivalent in increments of 0.25 diopters. ΔAL (mm): Represents the change in axial length in millimeters. ΔCA (D): Indicates the change in cornea astigmatism in diopters. SE Baseline (D): Baseline refractive spherical equivalent in diopters. AL/CR: axial length-corneal radius ratio. AL Baseline (mm): Baseline axial length in millimeters.

Adjusted model 1: AGE + SEX.

Adjusted model 2: AGE + SEX + AL/CR.

Adjusted model 3: AGE + SEX + AL/CR + SE Baseline, AL Baseline.

## Discussion

Our analysis suggested that baseline CA and ΔSE were significant predictors of CP progression. The magnitude of ΔSE had a positive correlation with CP progression, with larger changes associated with higher odds. In contrast, ΔAL did not appear to be a significant predictor in this data set.

Effective myopia prevention and control strategies include orthokeratology, low-concentration atropine drops, multifocal soft contact lenses, specially designed myopia control frames, and increased outdoor activities. Recently, specially designed frames have been getting more attention^14^. These lenses are popular because of their convenience, minimal side effects, and lack of age restrictions. The myopia control mechanisms of these lenses include peripheral defocus, peripheral image dispersion design, and peripheral low-contrast design. Clinical trials have shown that newer specially designed spectacle lenses that incorporate multiple segments, lenslets, or diffusion optics have good efficacy^7,15–19^.

In view of the impressive myopia control results reported, parents and optometrists who manage myopia with specially designed spectacles are concerned about the increase in myopia and the growth of astigmatism.

Our study is a real-world observational study with retrospective inclusion of clinical data. Due to the record keeping requirements of the refractive development archive, participants underwent cycloplegic refraction both before and after one year, with a complete record of the main examination result parameters.

We observed a myopia progression in ΔSE ((IQR) 0.25 (0.00, 0.75) D) and in ΔAL ((IQR) 0.24 (0.11, 0.38)) mm in this study. Determining the treatment effect using AL and SE as criteria resembled that of another real-world study^11^. In that study, not all results were obtained using cycloplegic refraction. The normalized annual changes in SER and AL in DIMS wearers were significantly smaller than those in SV wearers (DIMS; SER change vs. AL changes; −0.38 ± 0.32 D vs. 0.22 ± 0.16 mm. SV; −0.45 ± 0.41 D vs. 0.29 ± 0.20 mm, *P* < 0.05). Average axial elongation and myopia progression after 1 year were 0.17 (95% CI 0.13–0.20) mm and − 0.27 (95% CI − 0.36 to − 0.18) D in the DIMS group and 0.30 (95% CI 0.26–0.33) mm and − 0.55 (95% CI − 0.64 to − 0.47) D in the SV group in another recently RCT study^16^. The small differences in these studies may arise from whether cycloplegic refraction was performed or not.

Previous studies have shown good results in myopia control, but none have examined whether CP changes significantly during myopia control with specially designed myopia control frames. While previous RCT(randomized controlled trial)s for DIMS lens and real studies were only concerned with AL and SE, our study was also concerned with ΔCP based on the needs of parents and optometrists in a real clinical work environment. The results illustrated that 73.18% of the CP remained unchanged or even decreased individually. Parents and optometrists should not be concerned about the progression of subclinical CP during myopia control.

Our findings suggested that CP progression was associated with ΔSE and baseline CA in DIMS lens wearers. Similar results have been seen in young people with myopia^20^. Stratified analyses showed that the results remained robust across age and sex groups and different initial refractive errors.Age groups as proxies for biologigal, developmental, psychological, and social differences^21^. In our study, age groups were categorised as 6–9 and 9–12 years old, corresponding to grades 1–3 and grades 4–6, respectively, for Chinese primary school students. In China, the corresponding level of academic learning stress increases for students in grades 4–6. Therefore, through this categorization, we observe whether the occurrence of this type of risk is different under different age factors.

The main predictors of astigmatic change in DIMS spectacle wearers were ΔSE (OR = 1.73,1.32–2.27), and baseline CA (OR = 1.17,1–1.36), suggesting that optometrists should consider such two main correlations when explaining the possible causes of astigmatic change to parents. Additionally, more attention was paid to the use of myopia prevention and control measures with SE as the outcome, especially the combined use of multiple myopia prevention and control methods, to avoid an increase in ΔCP. Previous studies have shown that pharmaceutical interventions^22,23^ and light therapy^24^ may be more effective when combined with DIMS. Therefore, parents should be advised to use these in combination. However, attention must be paid to ΔCP if the DIMS lens wearer has high CA during the early baseline examination, even when various myopia prevention and control methods are utilized.

Previous study have shown that DIMS spectacle lenses can bring the AL growth rate of myopic children to the level of physiological AL growth rate^9^. Notably, we did not observe a significant positive correlation between ΔAL and CP progression. Unlike ΔSE, ΔAL is an important objective and convenient indicator for assessing the eye development patterns of children and adolescents and predicting the occurrence and development of myopia. However, individuals of different ages and varying myopic progress show different AL. Therefore, it is speculated that this may be the reason for the lack of significant correlation between ΔCP and ΔAL in this study.

On the one hand, The increase in children’s AL is relevant to their height increases. The faster their height increases, the faster their AL increases. Guangzhou twin eye study^25^ also discuss this connection.on the other hand, The principle function of defocus spectacles is to create myopic defocus on the peripheral retina while concurrently providing distance vision correction in the central zone.CP mainly shows the difference between two different meridian parameters. Due to the equivalence of different meridian defocus, it is possible that the CP is not related to defocus.It is hypothesized that CP may not be related to the defocus mechanism, whereas AL is only related to the value of the measured change in data in the middle portion of the retina. Therefore, in our study, It is speculated that ΔCP was not related to ΔAL.

Astigmatism is a refractive error that must be focused on in clinical practice. Total astigmatism results from the combined effect of corneal astigmatism and residual astigmatism of the eye. Optometrists and parents are always more concerned with CP progression in terms of astigmatism assessment, mainly because this relates to the lens form and appearance of edge thickness. Since there is the potential for offsetting or synergistic increases in AL and corneal astigmatism^13^ change in astigmatism should more often only consider ΔCP. This could also explain why ΔAL was not correlated with ΔCP in our study.

Previous studies have mainly focused on ΔSE and ΔAL as the main endpoints, but our study suggests that ΔCP can also be one of the main research objectives. The effects of myopia prevention and control can be quantitatively and qualitatively calibrated in the astigmatism dimension if better explored.A latest reference also suggests that spectacle lenses with a peripheral defocus design may increase astigmatism in myopic children^26^. Use of CP progression as a Focused Assessment Indicator.

## Conclusion

Parents and optometrists need not be concerned about the progression of subclinical CP in myopia control when using defocus incorporated multiple segments lenses.

In myopia management, corneal astigmatism (CA) measurement should be a routine examination for children and adolescents, especially those with large initial CA.

Follow-up is required for those diagnosed with myopia, and a large change in spherical equivalent (SE) is usually indicative of a large change in CP.

CP progression can be used as a secondary endpoint and observational indicator in studies on the effectiveness of clinically related myopia prevention and control of spectacle lenses.

## Limitations

Our study had several limitations. First, this was a single-center retrospective study, and it is thus susceptible to potential selection bias. Secondly, the small sample of CP progression and no control group in singer vision group of this study are key limitations.

Another limitation is the duration of the wear time. Although we found statistically significant effects after just one year of DIMS lens wear. A longer study would enable us to comment on the cumulative effects of the DIMS lens.

There was some confounding bias and selection bias in our study. We recognize it and will improve them in the future through Multi-center and prospective trials.

## Data Availability

The current study are available from the corresponding author on reasonable request.
